# Factors affecting community participation in drone-based larviciding using *Bacillus thuringiensis* var. israelensis (Bti) for bio-control of malaria vectors in Rwanda

**DOI:** 10.1186/s12936-025-05310-z

**Published:** 2025-03-01

**Authors:** Dunia Munyakanage, Elias Niyituma, Alphonse Mutabazi, Xavier Misago, Clarisse Musanabaganwa, Eric Remera, Eric Rutayisire, Mamy Muziga Ingabire, Aimable Mbituyumuremyi, Mathew Piero Ngugi, Elizabeth Kokwaro, Domina Asingizwe, Emmanuel Hakizimana, Claude Mambo Muvunyi

**Affiliations:** 1 Malaria and Other Parasitic Diseases Division, Rwanda Biomedical Center, Ministry of Health, Kigali, Rwanda; 2 Research, Innovation and Data Science, Rwanda Biomedical Center, Ministry of Health, Kigali, Rwanda; 3 Rwanda Biomedical Center, Ministry of Health, Kigali, Rwanda; 4 Charis Unmanned Aerial Solutions Ltd, Kigali, Rwanda; 5 Department of Zoological Sciences, Kenyatta University, Nairobi, Kenya; 6 EAC Regional Centre of Excellence for Vaccines, Immunization, and Health Supply Chain Management, College of Medicine and Health Sciences, University of Rwanda, Kigali, Rwanda

**Keywords:** Malaria, Larviciding, Drones, Community, *Bacillus thuringiensis* israelensis, Rwanda

## Abstract

**Background:**

Malaria remains a significant health issue in Rwanda. Primary malaria prevention methods include insecticide-treated nets and indoor residual spraying as core interventions. Mosquito repellents, larval source management (LSM), and housing improvement are recommended as supplemental vector control methods. A 2020–2021 study in rice field habitats of peri-urban of Kigali City successfully evaluated the entomological and epidemiological impacts of drone-based larviciding using *Bacillus thuringiensis* var. israelensis (Bti).

**Methods:**

The present study employed a concurrent mixed-methods design to assess community knowledge, perception, acceptance, and willingness to participate in drone-based larviciding for malaria control in Kigali City. A total of 248 respondents participated in the quantitative survey interviews while five focus group discussions (FGDs), each comprising 10–12 participants, were conducted. Quantitative data were analysed using SPSS and R software, with logistic regression applied to identify factors influencing community participation. Qualitative data were manually coded and analysed thematically to complement the quantitative findings.

**Results:**

Participants showed widespread knowledge of malaria transmission and prevention, with high awareness of the importance of larviciding. A strong support of 96.4% expressed willingness to accept drone-based larviciding, including financial and free labour support. Factors influencing willingness to participate include occupation in rice and vegetable farming and mining (95% CI − 3.053 to − 0.169, *p* = 0.029), mosquito exposure (95% CI − 5.706 to − 1.293, *p* = 0.004). Participants highlighted drone-based larviciding role in reducing mosquitoes and malaria risk and recommended it’s scaling up as a core component of integrated vector management (IVM).

**Conclusions:**

This study highlights strong community awareness and acceptance of drone-based larviciding, with its effectiveness in reducing mosquito abundance and malaria risks, along with the safety of Bti and drones. The findings advocate integrating drone-based larviciding into national malaria control strategies by enhancing community education, building local expertise, and adopting innovative financing mechanisms for scalability and sustainability.

## Background

Malaria remains a significant public health challenge in Rwanda, being one of the leading causes of morbidity and mortality [1]. Current malaria vector control strategies in Rwanda rely heavily on insecticide-treated nets (ITNs) and indoor residual spraying (IRS) as the core interventions. Larval source management (LSM) is considered a supplemental intervention in malaria vector control policy and strategies. The additional and implemented malaria control strategies include malaria diagnosis and treatment, monitoring and evaluation, and social behaviour communication change [2].

Despite notable progress, challenges persist. An estimated 66% of households in Rwanda own at least one ITN, but only 34% meet the Ministry of Health’s target of one ITN per two persons. While 78% of individuals with access to ITNs use them, achieving the national goal of 85% accessibility remains a challenge [3, 4]. IRS intervention, implemented in 13 high-malaria-burden districts since 2019, have provided protection against malaria to approximately 39.5% of the Rwandan population [5]. However, the emergence of insecticide resistance among malaria vectors and the presence of residual transmission continue to undermine these interventions [6].

To achieve malaria elimination, attain and maintain high coverage of existing vector control interventions while integrating innovative tools and technologies is critical [7]. Among these innovations, larviciding with *Bacillus thuringiensis* var. israelensis (Bti) presents an advantage of reducing the abundance and density for both larval and adult stages of malaria vectors, subsequently reducing the transmission of *Plasmodium* spp. [8], addressing residual malaria transmission foci and contributing to insecticide resistance management. Unlike conventional chemical insecticides, Bti has a low potential for resistance development, and its efficacy has remained stable over time [9].

The World Health Organization (WHO) recommends the use of larviciding in areas where mosquito breeding sites are few, fixed, and findable, such as urban settings. However, traditional larviciding methods face logistical challenges, particularly in treating large, hard-to-reach areas [10]. Drone technology offers a novel solution to these challenges. Drones can be used for mapping potential mosquito breeding habitats and landscapes, as well as for dispersing larvicide products, making larviciding interventions more efficient and cost-effective [11].

Successful implementation of larviciding interventions, particularly those involving drones, requires active engagement and participation of local communities. Community-based biological control of malaria vectors using Bti has been shown to be feasible in Rwanda and other parts of Africa [2, 8]. However, research on community acceptance of drone-based larviciding remains limited, both in Rwanda and across Africa. Understanding factors that influence community perceptions, acceptance, and willingness to participate is critical for optimizing the effectiveness of these interventions. To date, no study in Rwanda has assessed community perceptions and acceptance of larviciding using drones, and existing data on this topic across the continent are scarce [7]. This study conducted for a consecutive 10-months of drone-based larviciding operations addresses this gap by evaluating community knowledge, perceptions, acceptance and willingness to contribute financially and labour-wise to the Bti-based larval source management using drone technology.

This study assessed community knowledge, perceptions, and acceptance of Bti-based larviciding using drones, as well as their willingness to contribute financially and through labour. The findings will guide the development of strategies for large-scale deployment of drone-based larviciding interventions. The entomological and malaria-related impacts of this intervention are presented in a companion publication [12].

## Methods

### Drone-based larviciding using bti

The larviciding intervention employed Bti in the form of VectoBac^®^ WDG, applied through drones supplemented with hand application using agricultural knapsack sprayers operated by farmers selected from rice and vegetable cooperatives. These manual methods were specifically used in areas inaccessible to drones, such as the edges of water channels and small water bodies. Larviciding operations were conducted bi-weekly over a 10-month period, from July 2020 to April 2021, with the objective of targeting malaria vector aquatic stages to effectively interrupt malaria transmission.

**Fig. 1:**
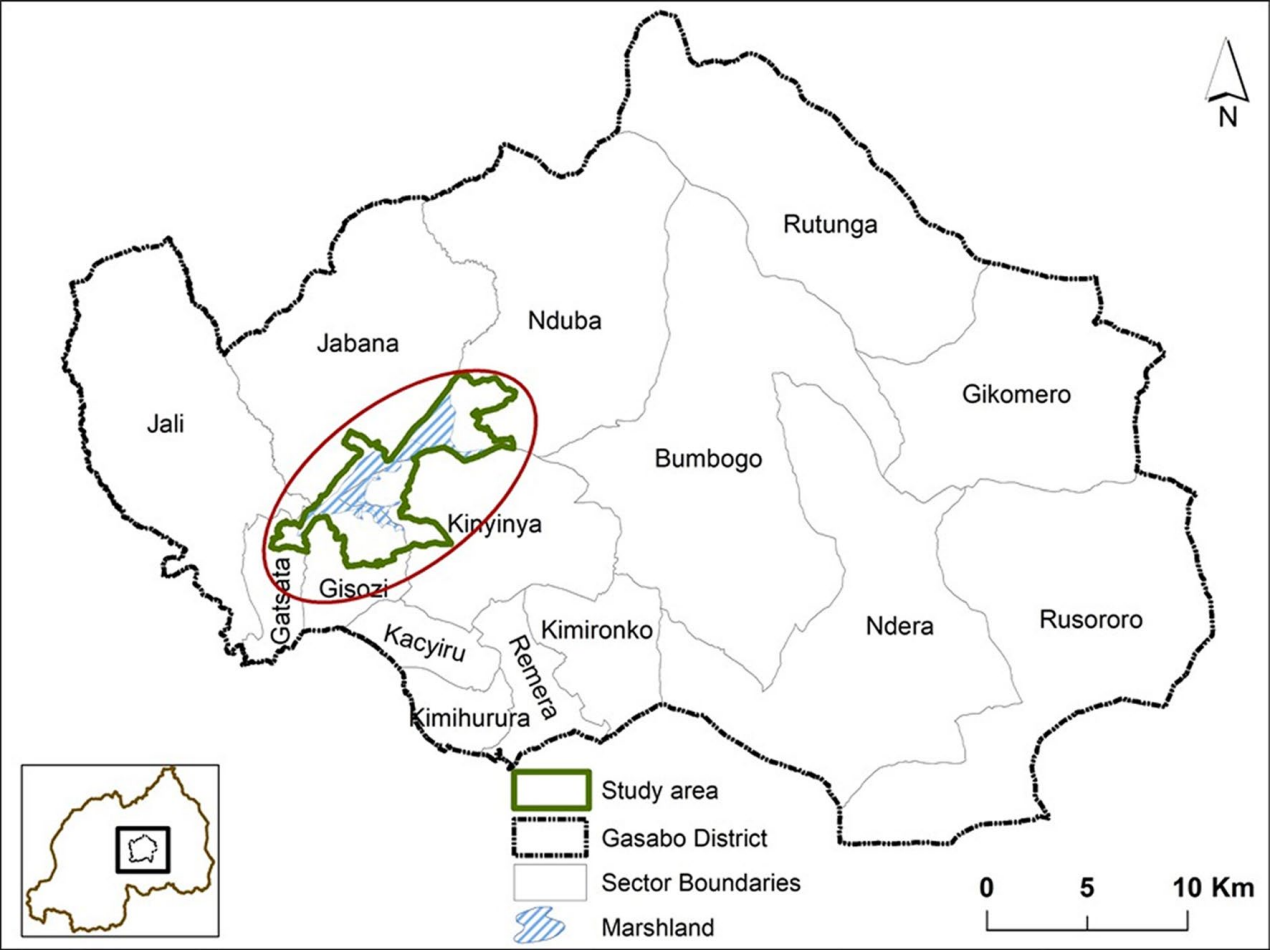
Location of the study site, Gasabo District, Kigali-City, Rwanda

The study area was divided into five marshland blocks, predominantly characterized by rice farming as the primary agricultural activity, along with vegetable cultivation and mining. For comparative analysis, the experimental area encompassed four blocks, covering a total of 336 hectares distributed across five administrative sectors of Gasabo District: Jabana, Gisozi, Gatsata, and Kinyinya (Fig. 1). The control area, located in Nduba sector, consisted of one block covering 78 hectares. To prevent spillover effects and ensure a clear distinction in the assessment of intervention impacts, the experimental and control areas were separated by a distance of over 5 km.

### Study area

This study was conducted in the northeastern of Gasabo District, located at 9.8 km from Kigali City. Despite being one of the districts in the capital city, Gasabo District has only 16% of its area developed as urban, with the remaining 84% classified as rural [13]. Gasabo District is situated at an elevation ranging between 1456 and 1800 m above sea level with sloping basins and valleys. It receives an annual average rainfall of 927 mm, with two rainy seasons alternating with two dry seasons. The rains occur from March to May and then from September to November. The dry season appears from December to February and from June to mid-September. The temperature varies between 17 and 28 degrees Celsius [14]. The majority of the population aged sixteen and above is employed in agriculture (31%), trade (17%), and government (11%). The remaining population works in sectors such as mining and quarrying, manufacturing, construction, transport and communications, recreation and tourism, and other services [15]. Malaria is highly endemic in the Gasabo District, with *Anopheles gambiae *sensu lato (*s.l.*) as the primary vector according to the 2022/2023 annual report (unpublished data) of the Malaria and Other Parasitic Diseases Control Division of the Rwanda Biomedical Center (RBC).

Due to the presence of complex marshlands in the study areas where stagnant water is abundant, irrigated rice farming is an important agricultural activity, with two annual farming cycles. The first is extended from July to December and the second from January to June. For the first 3 months of rice cultivation (July to September and January to March), watering practices of the rice fields generate the primary source of mosquito breeding sites. Additional types of mosquito breeding sites frequently encountered in the study areas are represented by intercrop water drains, mining pits and puddles, water dams used for small-scale irrigation, stagnant water in peri-domestic pits or trenches, and water in various used or unused containers. These types of breeding sites are primarily found and more productive after the rainy season [16–18].

### Study design

This study employed a concurrent mixed-methods design to assess community knowledge, perceptions, acceptance, and willingness to participate in drone-based larviciding for malaria control. The aim was to evaluate how various factors influence community participation in this novel approach to malaria control.

The quantitative component involved structured interviews using a questionnaire to gather data on participants’ knowledge of malaria, knowledge about larval source management using Bti with application done by drones supplemented by hand sprayers, and perceptions of Bti’s safety and effectiveness. The survey also examined participants’ willingness to contribute physically and financially to larviciding efforts. This cross-sectional survey provided a snapshot of community perspectives at a specific point in time.

The qualitative component involved focus group discussions (FGDs) with farming and mining cooperative members to explore community perceptions, challenges, and recommendations regarding the use of drones for larviciding. These discussions offered in-depth insights into how participants understood malaria transmission and the feasibility of drone-based interventions.

This mixed-methods approach provided a thorough understanding of the community’s knowledge, perceptions, and willingness to support drone-based malaria control efforts.

### Study population and sampling

**Fig. 2:**
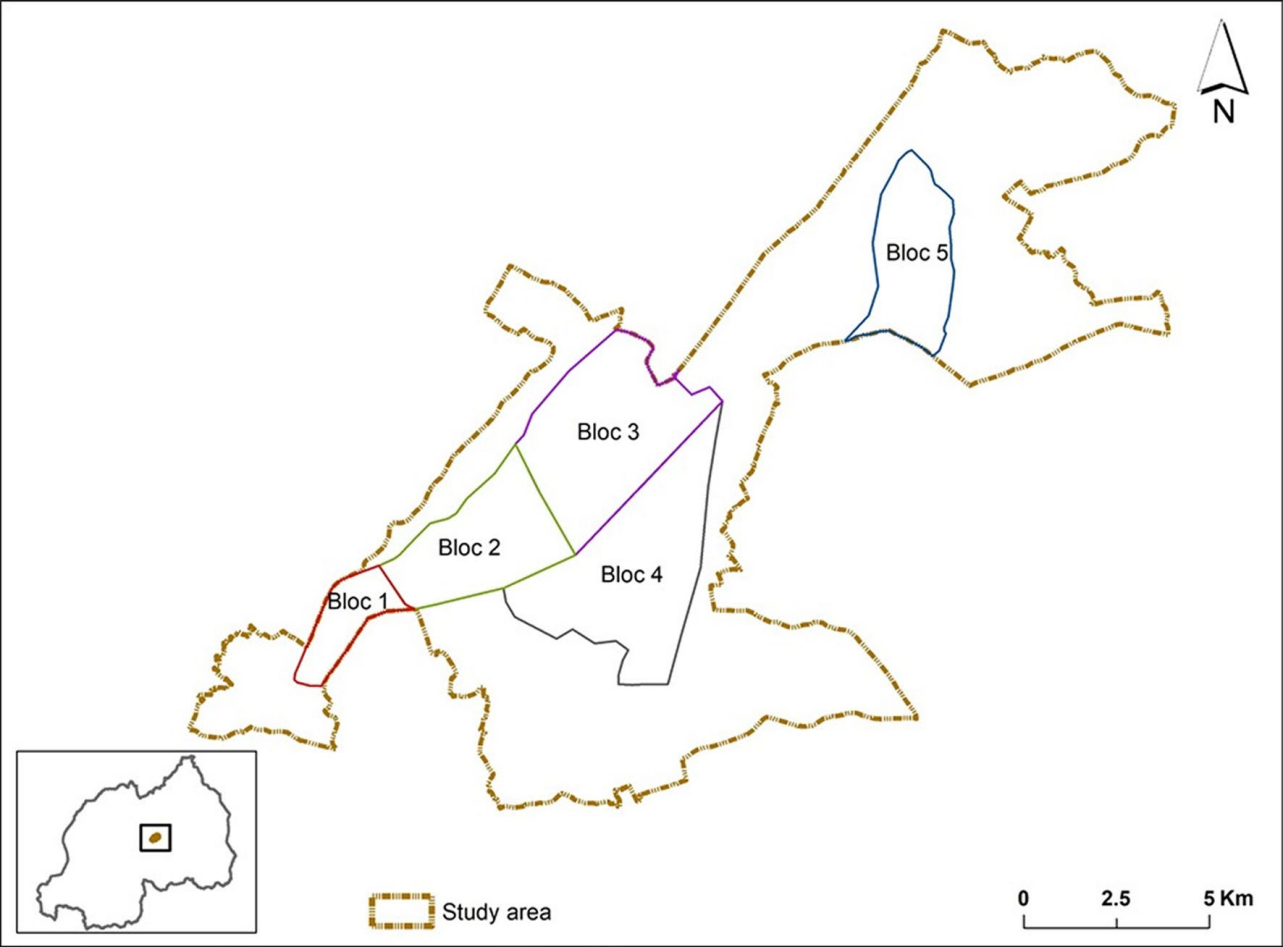
Map of the Study area displaying blocs of intervention and control arms

Participants in the study were drawn from four cooperatives/groups (CODAMUGA, CORIKA, COWABIGA, and MINES) located within the intervention and control areas, collectively comprising 699 members. They were categorized into three distinct groups: rice farmers, vegetable farmers, and miners. Rice farmers were distributed across Bloc 2 with 97 members, Bloc 3 with 110 members, Bloc 4 with 72 members, and Bloc 5 with 232 members. Vegetable farmers were represented in Bloc 1 with 80 members and Bloc 4 with 98 members. Miners, located in Bloc 4, consisted of 10 members (Fig. 2).

The sample size was determined using Yamane’s formula [19] for sample size calculation, applying a confidence level of 95%, a margin error of 5%, and an assumed population proportion of 50% to maximize variability. This calculation yielded a minimum required sample size of 248 participants. To ensure equitable representation of all cooperatives/groups, activities, and areas, the sample was proportionally distributed across the five blocs within the marshland (intervention and control) and the four cooperatives/groups based on their respective member sizes. For example, Bloc 5, with 232 members (33% of the total population), contributed the largest sample size of 82 participants, while the miners in Bloc 4, consisting of 10 members, contributed 4 participants, representing 1% of the population. Participants were randomly selected from the membership lists of their respective cooperatives/groups.

Qualitative data was collected through five heterogeneous focus group discussions (FGDs), with each FGD representing one bloc of marshland. Participants were recruited from the four cooperatives/groups of vegetable farmers, rice farmers, and miners. To ensure diverse responses, 10–12 participants were included in each FGD, totaling 54 participants in the study.

### Data collection instruments and measurement

The survey instrument and focus group discussion (FGD) guide were partly based on a structured questionnaire adapted from a previous Rwandan study on community-based biological control of malaria mosquitoes using Bti [2]. The original questionnaire was revised to fit the specific objectives of this study. After piloting in Blocs 1 and 2, revisions were made before full-scale data collection. All surveys and FGDs were conducted in Kinyarwanda and later translated into English.

### Data collection procedures

#### Quantitative data

A structured questionnaire developed using KoBoToolbox was used to conduct interviews with participants from different blocs between March 08 and March 19, 2021. KoBoToolbox, a digital data collection platform, enabled real-time recording and synchronization of responses to ensure data accuracy and reduce input errors. A team of 20 trained data collectors working with investigators, was trained on how to use the KoBoToolbox that was installed on their tablets to facilitate efficient data collection. The training focused on using the platform, administering the questionnaire, and adhering to ethical research practices, including ensuring informed consent and maintaining participant confidentiality**.** The survey covered demographics and knowledge of malaria transmission. Participants view on larval source management (LSM) using Bti applied by drones and hand sprayers, and perceptions of Bti’s safety and effectiveness. Finally, participants were asked about their willingness to contribute to larviciding efforts. Data collection was conducted on-site at farms or mining locations to gather firsthand insights and ensure contextual relevance, while ensuring gender balance by including both male and female participants.

#### Qualitative data

The qualitative component was conducted right after completing the quantitative survey, from March 22 to April 2, 2021. Therefore, the latter did not influence the former. Investigators led focus group discussions with cooperative members and their leaders, using a focus group guide that addressed topics closely aligned with those of the quantitative interviews. The discussions focused on awareness of malaria transmission, risk factors, prevention methods, larval source management (LSM), the efficacy and safety of Bti, and the use of drone technology for mapping and applying Bti. Participants shared their perceptions of the impact of larviciding on malaria transmission and their willingness to engage in drone-based larviciding activities. They also discussed their relationships with larval monitoring teams, sprayers, and drone pilots, along with challenges and recommendations related to the scalability of drone-based larviciding efforts. To ensure gender balance, heterogeneous groups were formed and both male and female participants took part in the discussions. A total of five focus groups were held across the five research blocs, each consisting of ten to twelve participants and lasting approximately one to two hours. The first author (DM) and his assistant took notes during the discussion.

#### Ethical considerations

The Rwanda Biomedical Centre, through its Division of Research, Innovation, and Data Sciences, reviewed and approved this study under reference number 225/RBC/2020, granting the necessary clearance for its conduct. Prior to each interview and discussion, written informed consent was obtained from participants in the presence of their cooperative leaders. However, the leaders did not exert any influence on the participants’ decision to take part. All the participants were men and women above the age of 18 years old. Participants were assured that their personal information would remain confidential and would not be included in transcripts. Lastly, participants were assured that participation in the study was entirely voluntary and that they could withdraw from the interview and discussions at any moment.

#### Data analysis

For quantitative data, following data collection using the programmed questionnaire, additional data inspection, cleaning, pre-processing, and transformation, where necessary, and were conducted to ensure a clean, useful, valid data set ready for analysis. Summary statistics including descriptive statistics and logistic regression models were conducted using SPSS Version 25 and R version 4.3.3. The threshold for statistical significance was set at a *p*-value of < 0.05 with 95% confidence intervals.

For the qualitative data and to ensure trustworthiness [20], data were gathered through focus group discussions (FGDs) conducted by the principal investigator with support from other investigators. During these sessions, the principal investigator personally took detailed notes to complement the quantitative data capturing participants’ statements as accurately as possible. These notes were subsequently translated into English by the principal investigator, ensuring that the exact meaning and phrasing of the participants’ contributions were preserved.

The analysis process was designed to align with the study objectives, combining both deductive and inductive approaches. A coding framework was developed based on pre-defined themes derived from the FGD questions, which covered topics such as community perceptions of malaria causes, safety concerns regarding larviciding, the role of drones in mapping and spraying, acceptance of the intervention, willingness to participate and suggestions for scaling up drone-based larviciding interventions using Bti. The framework also allowed flexibility for the identification of emergent themes that were not initially anticipated.

Translated notes were systematically coded using the established framework. Two independent investigators reviewed the coded data to ensure consistency, reliability, accuracy and alignment with the study objectives. Any discrepancies in coding were resolved through discussion, and the framework was adjusted to incorporate shared insights. Iterative analysis was conducted to refine the themes and sub-themes, ensuring that the coding framework captured all relevant insights.

The final themes were discussed and agreed upon by all co-authors to achieve consensus and ensure alignment with the study objectives. Representative quotes were selected to illustrate key findings, ensuring they accurately captured the diversity of participants’ perspectives. These quotes were reviewed and cross-checked against the original notes to maintain the integrity of the data.

## Results

### Quantitative results

#### Socio-demographic characteristics of respondents

A total of 248 respondents participated in the quantitative survey (Table 1). All the interviews were conducted with the members of rice and vegetable farming and mining cooperatives. The mean age was 47.02 years (SD 12.2), and the mean number of children under five per household was 1.14. Primary education was the highest level of formal education, with 56.9% of respondents. The interviews had a higher percentage of male respondents (53.2%) than females (46.8%), with a mean of 5.2 household members. The majority (73%) of the respondents reported being rice farmers as their occupation, while 25.4% were vegetable farmers, and the remainder (1.6%) were miners (Table 1).

**Table 1:** Characteristics of study respondents

Variable	Response	Frequency	Proportion (%, SD)
Gender	Male	132	53.2%
	Female	116	46.8%
Age group	20–34	44	17.7%
	35–50	105	42.3%
	≥ 51	99	39.9%
Education level	None	53	21.4%
	Read only	20	8.1%
	Primary	141	56.9%
	Vocational training	6	2.4%
	Secondary	23	9.3%
	Higher	5	2.0%
Occupation	Rice farmers	181	73%
	Vegetable farmers	63	25.4%
	Miners	4	1.6%
Years worked (Rice farming)	0–5	60	33.1%
	6–14	46	25.4%
	≥ 15	75	41.4%
Years worked (Vegetable farming)	0–5	35	55.6%
	6–14	21	33.3%
	≥ 15	7	11.1%
Years worked (Mining)	0–5	2	50%
	6–14	1	25%
	≥ 15	1	25%
Marital status	Married/living together	201	81%
	Never married/single, separated, divorced, widowed	47	19%
Household characteristics	Mean number of people	5.2	SD 2.3
	Mean number of children under five	1.14	SD 1.3

#### Knowledge and practices regarding malaria and larviciding

The study findings revealed widespread knowledge among participants about the causes and symptoms of malaria. Of the total sample of 248 respondents, 197 (79.4%) identified mosquitoes as the vector of malaria parasites, 72 (29%) could even mention the specific sex and genus of the mosquito (female *Anopheles*) responsible for the transmission of malaria, 239 (96.4%) cited fever as a malaria symptom, 182 (73.4%) believe that their irrigation, mining practices create mosquito breeding sites. In accordance with their knowledge of mosquito aquatic stages, 71 (28.6%) reported having seen mosquito larvae, and the majority 157 (63.3%), indicated stagnant waters as a frequent mosquito breeding habitat. Overall, respondents were aware of malaria vector control methods in their community, with 101 (40.7%) citing mosquito nets, 96 (38.7%) citing Indoor Residual Spraying (IRS), and the majority (more than 70%) citing larval source management (LSM), with larviciding cited by 175 (70.6%) and removal of stagnant water by 179 (72.2%) (Table 2).

**Table 2:** Knowledge of participants related to malaria transmission and vector control

Variables	Categories	Frequency	Percentage
Causes of malaria	Bite from a mosquito	197	79.4%
	Bite from a female *Anopheles* mosquito	72	29%
Frequency of self-reported mosquito bites	Almost never	49	19.8%
	Once in a while	92	37.1%
	Often	59	23.8%
	Very Often	48	19.4%
Symptoms of malaria	Fever	239	96.4%
	Headache	171	69.0%
	Chills	102	41.1%
Ways activities of irrigation farming, rice cultivation, and mining impact on malaria transmission	Not at all important	66	26.6%
	Minor importance	46	18.5%
	Important	83	33.5%
	Very important	53	21.4%
Knowledge about mosquito aquatic stages	Have ever seen a mosquito larva	71	28.6%
Knowledge about mosquito breeding sites	Stagnant waters	157	63.3%
	Nearest forest	23	9.3%
	Pit toilet	9	3.6%
	Bushes surrounding the house	67	27%
	House items that hold water	39	15.7%
Malaria vector control methods	Use of mosquito net	101	40.7%
	Indoor Residual Spraying (IRS)	96	38.7%
	Larviciding	175	70.6%
	Removing stagnant water	179	72.2%
	Removing items that can hold water	73	29.4%
	Housing improvements (Windows and doors screening, walls, roof, floor, …)	23	9.3%
	Removing bushes surrounding the house	129	52%
	General hygiene of the house	20	8.1%

#### Attitudes and awareness towards drone based Bti application supplemented by hand sprayers

Table 3 presents results on awareness towards drone based Bti application supplemented by hand sprayers, 99.2% of respondents indicated being aware of the ongoing Bti larviciding campaign in their marshlands, 93.5% of participants recognized the importance of using drones in larviciding, among those who acknowledged the importance of drones, 69% cited the quick check of water bodies as a reason, 42.3% mentioned efficiently dispersing larvicides product, 22.6% believed in reducing operational costs, 52% highlighted reaching places inaccessible to hand sprayers as a significant factor (Table 3).

**Table 3:** Awareness towards drone based Bti application supplemented by hand sprayers: Dichotomus responses

Variable	Percentage Responding Yes (%)
Awareness of the ongoing Bti larviciding campaign in their marshland	99.2
Importance of using drones in larviciding	93.5
Reasons for using drones in larviciding:	
Quick check of water bodies	69
Efficiently dispersing larvicides/Bti	42.3
Reduce operation costs	22.6
Reach places inaccessible to hand sprayers	52

#### Attitudes towards larvicide and drones’ safety

Table 4 presents findings on the perceived safety of larvicides, particularly Bti, and the use of drones across various contexts and groups. The table summarizes responses on a 4-point scale, ranging from “Not Safe” to “Very Safe.” A significant percentage of the respondents, 35.1% considered Bti very safe for its impact on agricultural products, while 25.8% rated it as safe. Regarding the consumption of agricultural products treated with larvicides, 33.9% viewed larvicides as very safe. In terms of Bti’s safety for farmers and miners, 35.1% rated it very safe, with 37.1% considering larvicides very safe for hand sprayers. When asked about the safety of larvicides for other organisms beyond mosquito larvae, 29.8% considered them very safe (Table 4).

**Table 4:** Attitudes towards larvicide and drones’ safety: rating scale responses

Statement	Not safe (%)	Somewhat Safe (%)	Safe (%)	Very safe (%)
Safety impact of the larvicides (Bti) on agricultural products such as rice, vegetables, …	31	8.1	25.8	35.1
Safety impact of the larvicides (Bti) on agricultural products consumption such as rice, vegetables, …	23.4	9.7	33.1	33.9
Safety impact of larvicides (Bti) on farmers and miners	29	9.7	26.2	35.1
Safety impact of larvicides (Bti) on hand sprayers	15.7	13.7	31.9	37.1
Larviciding safety on living of other organisms such as fish, dragonflies, frogs etc.…	29.4	14.5	26.2	29.8
Safety of drones use in larviciding	10.9	4	22.2	60.9

Table 5 presents respondents’ perceptions regarding the effectiveness of larviciding with Bti, in reducing mosquito abundance, density, and malaria risk. Participants responded on a grading scale from “Not all” to “Extremely”. The majority of respondents, 45.6% rated larviciding as extremely effective in reducing mosquito abundance, and 36.3% considered it moderately effective, similar trends were observed, with 50% of respondents rating larviciding as extremely effective in reducing mosquito density while 35.1% considered it moderately effective, respondents exhibited confidence in the effectiveness of larviciding in reducing malaria risk, with 47.6% rating it as extremely effective and 39.9% considering it moderately effective (Table 5).

**Table 5:** Perceived effectiveness of larviciding: grading responses

Effectiveness of larviciding (Bti)	Not at all (%)	Slightly (%)	Moderately (%)	Extremely (%)
Reduction of mosquito abundance	6.5	11.7	36.3	45.6
Reduction of mosquito density	4.4	10.5	35/1	50
Malaria risk reduction	3.2	9.3	39.9	47.6

#### Acceptance of larviciding intervention

The study findings, as presented in Table 6, underscore a notable level of community awareness regarding the role of their agricultural (rice and vegetable farming) and mining activities in facilitating mosquito reproduction, with 83.9% of participants acknowledging this fact. Moreover, the data reveal a high level of acceptance of larviciding intervention among study participants, with 96.4% of respondents expressing willingness to accept such intervention. Logistic regression analysis identified key predictors of this high level of acceptance. These included occupations involving proximity to mosquito breeding sites, such as rice and vegetable farming and mining, frequent mosquito exposure, beliefs in the effectiveness of larviciding, and perceptions of stagnant water as mosquito breeding sites. In conjunction, the data indicates that a majority of participants (81.0%) is willing to accept less profit or dividend if their cooperatives contribute to the larviciding intervention. Furthermore, the findings indicate that a significant proportion of respondents, totaling 94%, were willing to allocate time for larviciding intervention, ranging from 30 min to more than 2 h biweekly (Table 6).

**Table 6:** Community acceptance and engagement in larviciding intervention: Dichotomous responses

Variable	Percentage responding yes (%)
Knowledge that participant’s activities promote mosquito reproduction	83.9
Acceptance of larviciding intervention	96.4
Willingness to physically and financially contribute to larviciding intervention	80.6
Willingness to accept reduced profits/dividends with cooperative’s contribution in larviciding intervention	81
Willingness to devote additional biweekly labour for larviciding intervention	
None	6
30 min	8.9
1 h	29
2 h	22.2
> 2 h	33.9

#### Exploring monthly willingness-to-pay for larviciding intervention

**Fig. 3:**
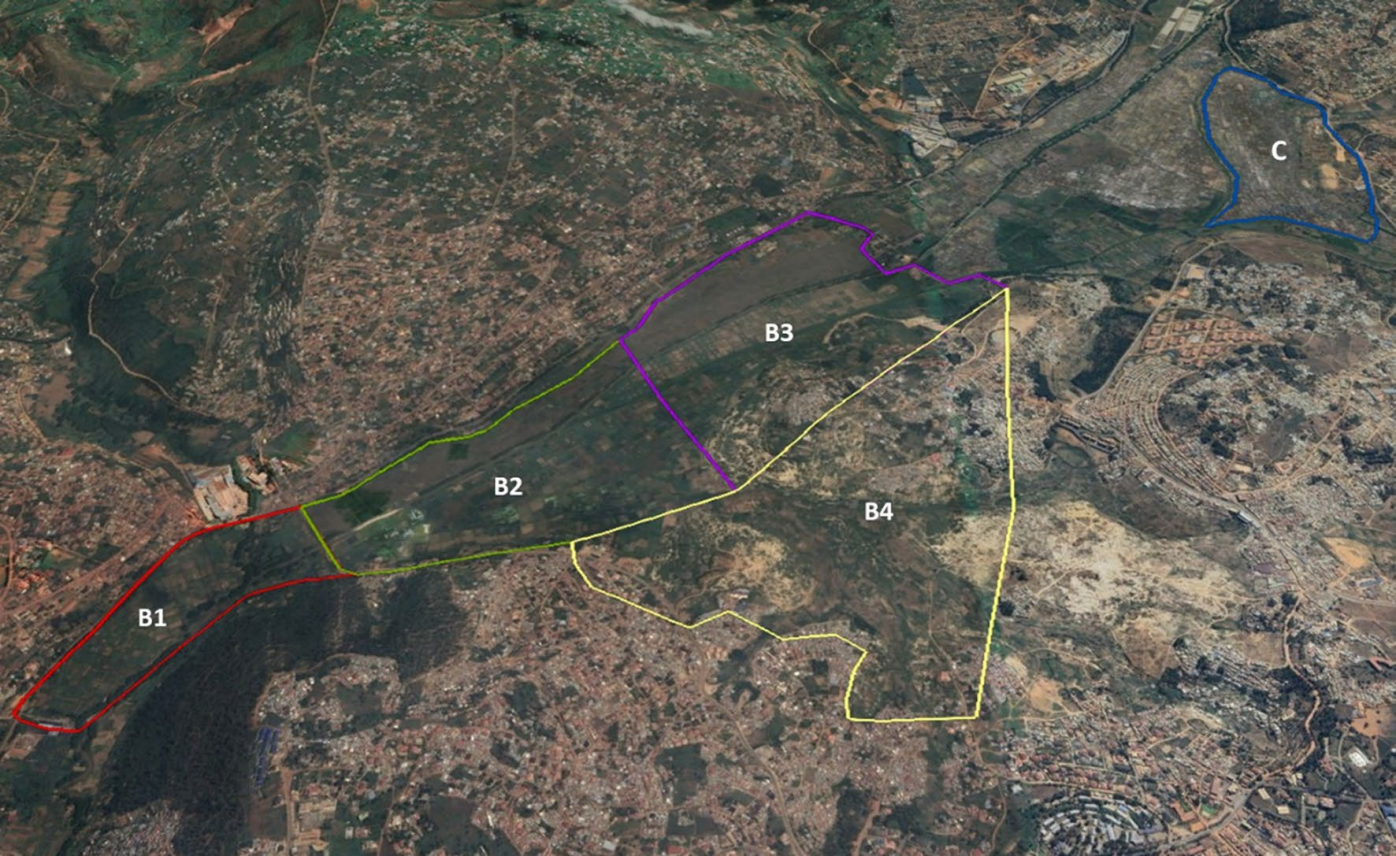
Aerial view of the study site featuring five blocs surrounded by human settlements

The data description analysis presents insights into the willingness of individuals to financially support larviciding interventions across various contribution scenarios, as outlined in Fig. 3. Approximately 25.0% of respondents reported facing financial challenges, indicating potential barriers to financial participation. Notably, the majority of respondents (35.9%) expressed willingness to contribute 500 RWF (equivalent to 50 cents US$), suggesting a significant level of community support for larviciding interventions at relatively lower contribution levels. Conversely, a smaller proportion of respondents indicated readiness to contribute at relatively higher monetary thresholds, with 13.7% and 4.8% expressing willingness to pay 1000 RWF (1 US$), 1500 (1.5 US$) and 2000 RWF (2 US$), respectively.

**Fig. 4:**
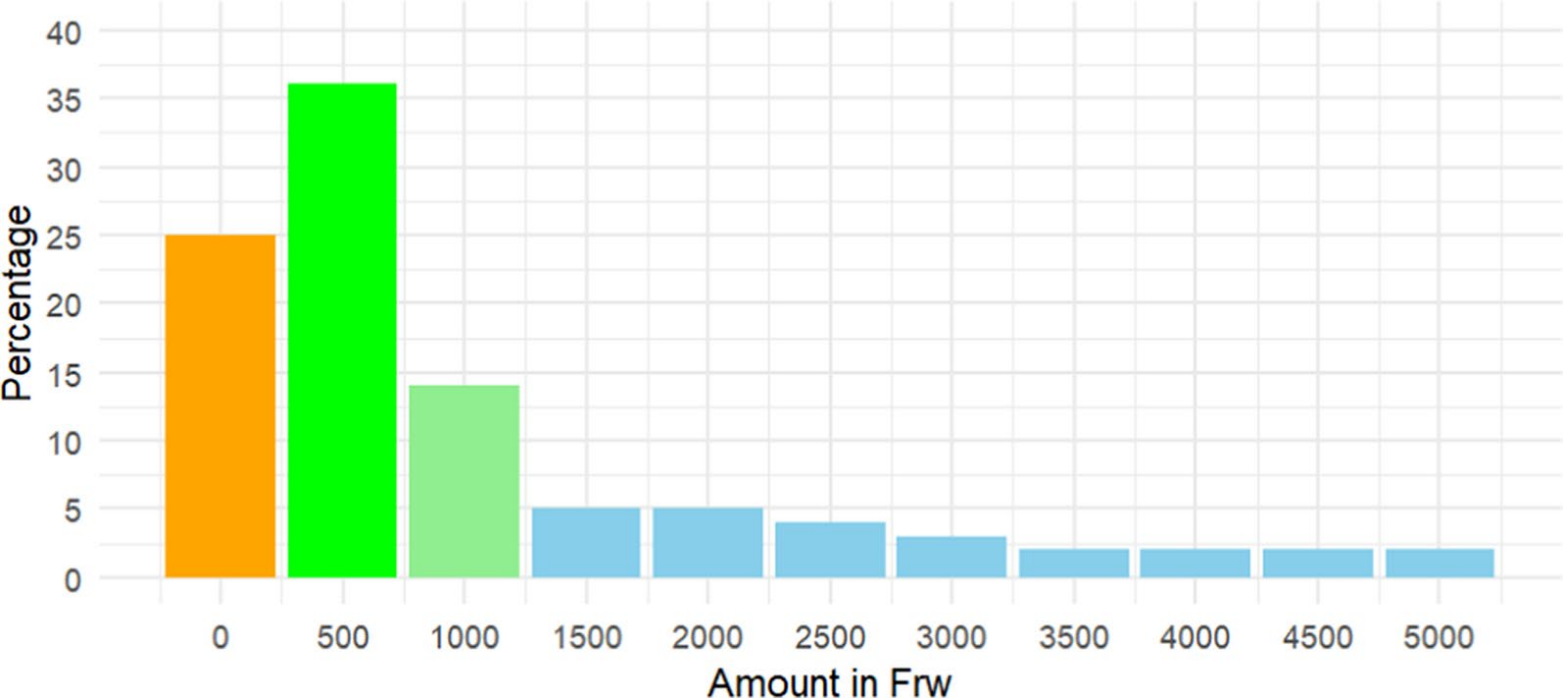
Willingness to pay for larviciding intervention, by contribution amount per month

The analysis further reveals a notable trend wherein the number of participants willing to pay decreases as the amount increases, ultimately culminating in only 2% of respondents opting for amounts of 4500 (4.5$) and 5000 RWF (5$) (Fig. 4). This decline in participation with increasing contribution amounts underscores a nuanced relationship between willingness to contribute and financial thresholds, suggesting potential barriers or limitations in engagement as contributions escalate.

The application of Winsorization technique to the willingness-to-pay, with 10% trimming from both ends, yielded a lower bound mean estimation of approximately 939.52 RWF (0.94$) contribution per month.

#### Factors associated with willingness to contribute to the drone based larviciding intervention

A logistic regression analysis was conducted to examine factors associated with willingness to contribute to the drone based larviciding intervention. The analysis included 248 participants, and the outcome variable was binary, indicating whether individuals were willing to physically participate and financially contribute or not. The results revealed several predictor variables that were significantly associated with willingness to contribute. Table 7 summarizes predictor variables along with their estimated coefficients, 95% confidence intervals (CI), and *p*-values (Table 7).

**Table 7:** Logistic regression analysis of predictors for willingness to participate and contribute to drone-based larviciding intervention

Predictor Variable	Coefficient	95% CI	z-value	*p*-value
Age	− 0.039	(− 0.105, 0.027)	− 1.133	0.257
Gender	− 0.845	(− 2.124, 0.433)	− 1.283	0.199
Main occupation in rice, vegetable farming, mining	− 1.611	(− 3.053, − 0.169)	− 2.185	0.029*
Number of years involved in activity (rice/vegetables farming, mining)	− 0.011	(− 0.080, 0.058)	− 0.304	0.761
Frequency of mosquito bites	− 2.543	(− 4.681, − 0.404)	− 2.335	0.020*
Number of household members	0.069	(− 0.274, 0.412)	0.393	0.695
Bite from a female Anopheles mosquito as a cause of malaria	− 1.895	(− 3.531, − 0.258)	− 2.114	0.035*
Bite from a mosquito as a cause of malaria	− 3.500	(− 5.706, − 1.293)	− 2.848	0.0044**
Stagnant waters as a source of mosquito larvae	− 6.025	(− 9.838, − 2.212)	− 3.030	0.002**
Reduce mosquitoes by removing all items that can hold water	− 1.338	(− 2.531, − 0.145)	− 2.182	0.029*
Reduce mosquitoes by removing bushes surrounding the house	− 1.475	(− 2.884, − 0.066)	− 2.047	0.041*
Reduce mosquitoes with the use of larviciding	4.220	(0.887, 7.554)	2.467	0.014*
Household member who suffered from malaria in the last 12 months	3.724	(1.802, 5.646)	3.794	< 0.001***
Belief that the use of drones in larviciding is safe	− 2.71254	(− 4.96013, − 0.46494)	− 2.251	0.024*
Awareness that participants’ activities favour mosquito reproduction	3.19596	(0.70912, 5.68280)	2.982	0.003**
Willingness to get less profit if cooperatives contribute to larviciding	5.49466	(3.50150, 7.48781)	5.430	< 0.001***

Willingness to Contribute (Y/N); N = (248). The *p*-values marked with an asterisk (*) denote statistical significance at the 0.05 level, ** indicates significance at the 0.01 level, and *** indicates significance at the 0.001 level. CI represents the 95% Confidence Interval. A CI that does not include zero indicates a statistically significant effect. The logistic regression model was fitted using a binomial family in R to assess predictors of willingness to contribute to drone-based larviciding. Statistically significant variables were retained for interpretation

Several factors were examined, including demographic variables such as age, gender, and occupation, as well as other contextual variables like participant’s mosquito exposure, perceived causes of malaria, environmental factors influencing mosquito reproduction, household experience with malaria, perceptions of drone safety, and awareness of mosquito reproduction activities.

Age, gender, duration of engagement in activities such as rice and vegetable farming, and household size, were found to lack significant associations with willingness to contribute. However, occupations, specifically in rice and vegetable farming, and mining, exhibited a statistically significant correlation with willingness to contribute, as did frequent exposure to mosquito bites. Notably, beliefs attributing malaria to mosquito bites, particularly from female *Anopheles* mosquitoes, were strongly linked to participants’ willingness to contribute. Perceptions regarding stagnant water as a breeding source for mosquito larvae, along with methods involving the removal of mosquito habitats and surrounding bushes, were positively associated with willingness to contribute. Moreover, belief in the effectiveness of larviciding emerged as a robust predictor of willingness to contribute. Households with recent malaria cases and increased awareness of mosquito reproduction activities were additional drivers that incrementally increased their willingness to contribute. Furthermore, perceptions of drone safety during larviciding operations and a willingness to forego personal profits from cooperatives for larviciding efforts exhibited significant associations with contribution willingness.

### Qualitative results

Qualitative findings complement quantitative results, emphasizing the community’s recognition of their role in creating mosquito breeding conditions through agricultural and mining activities. Participants expressed how larviciding interventions reduced mosquito nuisance, allowing them to work longer hours in fields. They also highlighted the drones’ role in improving efficiency and precision in larviciding operations.

In the focus group discussions (FGDs), several themes emerged regarding community knowledge and perspectives on malaria transmission, mosquito breeding sites, and the effectiveness of drone-based larviciding operations. These insights provide a deeper understanding of the community’s experiences, views, and recommendations regarding drone-based larviciding and malaria control. Each theme and related quotes are described in detail in the following section.

#### Community knowledge of malaria transmission and mosquito breeding sites

Among FGD participants, it was widely acknowledged that malaria primarily arises from mosquito bites. The mosquitoes breed in stagnant water bodies and subsequently enter homes to feed on individuals. In this example, a rice farmer, zone leader emphasized:

“*The primary cause of malaria is the bite of mosquitoes, which develop in stagnant water bodies. Subsequently, adult mosquitoes fly into people’s homes to feed on individuals*” *(FGD, Male, 51, Bloc 3*)

Another participant complemented this by stating that *“malaria is caused by mosquito bites from mosquitoes that breed in water bodies, especially when people do not sleep under SUPANET (mosquito nets)*” (FGD, Male, 50, Bloc 1).

#### Community insights on the safety of Bti larvicides on agriculture and biodiversity components

Participants were generally aware of ongoing drone-based larviciding activities that have been carried out over the past 6 months. Regarding their experiences, one participant, a rice farmer and team leader, noted:

*“We haven’t noticed any problems with our crops or those applying the Bti. At the beginning, we were a bit worried about any harm that might come from even tiny droplets of water mixed with Bti touching people. But as time went on, we realized there was nothing to fear. No issues whatsoever*” (FGD, Male, 31, Bloc3).

In the same line, another participant added*:*

*“The larvicide products, specifically this one, Bti, that is being used in our fields, cause no harm to either people or plants. As a vegetable farmer, I have noticed that since the larviciding activities started, our crops have remained healthy and unaffected. We can continue our farming activities without worrying about negative impacts on our harvests. Moreover, the safety of our workers is paramount, and I can confidently say that Bti poses no risk to their health. It allows us to manage mosquito populations effectively while ensuring that our agricultural practices remain sustainable and safe for everyone involved”* (FGD, Male, 35, Bloc 1).

No participants reported any adverse effects on crops or on people, including workers and farmers, underscoring high community acceptance of Bti use. However, participants did not comment on its impact on biodiversity, including non-target organisms such as pollinators or aquatic species.

#### Community insights on larviciding’s impact on mosquitoes

There was a consensus among different groups that before larviciding activities began, mosquitoes were of high nuisance, prompting farmers to head home before 3 PM due to mosquito bites. However, since the beginning of larviciding implementation, things have changed, farmers can now work in their fields well into the late evening without fear of mosquito bites. One male, vegetable farmer stated:

*“The mosquitoes that used to bother us have significantly decreased. Back when we were working in the fields, mosquitoes would bite us, and by around 3 PM, people would start heading home. But now, thanks to the changes, we can keep working in the fields well into the late evening without worrying about mosquito bites”* (FGD, Male, 41, Bloc 1).

A different participant supported this view, stating that:

*“spraying the marshlands has effectively reduced mosquito populations, but non-sprayed areas uphill still remain vulnerable”* (FGD, Male, 54, Bloc 5).

#### Community insights into larviciding’s effectiveness in reducing malaria risk

Amid discussions on the effectiveness of larviciding in reducing malaria risk, a majority of participants expressed high expectations, citing tangible improvements in reduced malaria cases among farmers and in their villages. One participant, a female rice farmer said*:*

*“Absolutely, larviciding has made a real difference in reducing malaria. In the past, before larviciding started, community health workers would see many malaria patients. But now, those numbers have decreased significantly. Even when we visit health centers, it’s rare to come across patients with malaria”* (FGD, Female, 64, Bloc 3).

Another participant affirmed this, noting*:*

*“The reduction of mosquitoes in homes near the spraying blocs has directly contributed to the noticeable decrease in malaria cases”* (FGD, Male, 54, Bloc 5).

#### Community perspectives on the importance of drones in larviciding operations

In FGD discussions regarding the importance of using drones in larviciding activities such as mapping and spraying, participants emphasized the critical role of drones in reaching inaccessible areas efficiently and swiftly completing the tasks. They highlighted the potential damage of crops when the hand sprayers walk in rice fields and praised drones for their precise mapping and aerial spray capabilities, facilitating targeted application of larvicides, a female rice farmer said*:*

“*Undoubtedly, it’s crucial to use drones. There are areas, especially in the middle of rice fields, where hand sprayers can’t reach on foot, but drones can access them efficiently. Drones also swiftly complete their tasks, unlike hand sprayers that rely on pumps. Furthermore, traversing rice fields on foot can potentially damage the crops. Additionally, drones equipped for mapping can precisely survey large areas, facilitating targeted application of larvicides”* (FGD, Female, 62, Bloc 5).

A similar perspective was shared by another participant*:*

*“Drones are especially effective in rice fields, where larger areas can be covered, but they may be less practical in vegetable fields due to the smaller and more scattered water bodies”* (FGD, Male, 36, Bloc 4).

#### Community perspectives on the importance of hand sprayers in larviciding operations

Regarding the importance of using hand sprayers in larviciding, participants in FGD discussions highlighted the need to complement drone usage with hand sprayers, especially in areas inaccessible to drones, such as the edges of water channels and small water bodies, one male participant noted that.

“*It is important to complement the use of drones with hand sprayers, especially in areas inaccessible to drones, such as the edges of water channels, small water channels or pits, and small water bodies”* (FGD, Female, 26, Bloc 1).

Another participant stated:

*Hand sprayers provide employment opportunities for individuals”* (FGD, Male, 53, Bloc 5).

#### Community awareness of mosquito breeding conditions linked to participants’ agricultural and mining activities

In FGD discussions, participants acknowledged their activities’ role in creating favorable conditions for mosquito breeding. Some respondents highlighted:

“*Stagnant water is where mosquito reproduction occurs, often found where people step and in poorly maintained channels where water doesn’t flow well.”* (FGD, Female, 36, Bloc 3).

Another one added.

*“Due to rice cultivation, mosquitoes breed abundantly”* (FGD, Male, 53, Bloc 2).

Adding to this, another respondent stated:

*“The water used in irrigation serves as breeding ground for mosquitoes”* (FGD, Male, 23, Bloc 1).

#### Community willingness to accept drone-based larviciding intervention

Participants in the FGD expressed their willingness to accept drone-based larviciding intervention in their fields without hesitation*,* one participant, a rice farmer highlighted*:*

*“We have no objections to larviciding intervention in our fields"* (FGD, Male, 41, Bloc 4).

Another FGD participant shared a similar sentiment, stating.

*“We have no concerns about applying larviciding in our fields; it poses no problem for us at all. In fact, we are fully supportive of its use to help control mosquito breeding and reduce the malaria risk in our community”* (FGD, Male, 40, Bloc 3).

As corroborated by the quantitative results, 83.9% of participants acknowledged the role of rice and vegetable farming in facilitating mosquito reproduction, while 96.4% expressed acceptance of larviciding interventions, highlighting a strong alignment between community awareness and proactive support for drone based larviciding intervention.

#### Community’s willingness to physically and financially contribute to drone based larviciding intervention

Concerning their willingness to contribute personally and financially to the larviciding intervention efforts in their fields, participants demonstrated proactive engagement. They conveyed a strong readiness to engage in the initiative, highlighting their eagerness to play an active role. This readiness was evident in their willingness to both contribute financially and physically participate in the larviciding intervention activities.

*“If we are asked to provide financial support and the contribution is small, we are willing to offer it. Even if it means volunteering our time and efforts for larviciding activities like spraying, we are ready to participate voluntarily”* (FGD, Male, 52, Bloc 4)*.*

Furthermore, participants recommended to involve their respective cooperatives, emphasizing the importance of collective action and community collaboration. One participant, male, vegetable farmer:

*“We can contribute financially, but the amount should not be substantial, perhaps around a thousand francs per month. Additionally, we are willing to participate physically and view it as community work. We also believe it’s important to discuss and involve our cooperative in this endeavor”* (FGD, Male, 50, Bloc 1).

Participants showed a strong willingness to contribute both physically and financially, emphasizing their readiness to dedicate time and provide modest financial support. They also highlighted the role of their cooperatives in promoting collective responsibility.

#### Community perspectives on the use of other existing malaria preventive measures alongside larviciding

Regarding the potential impact of larviciding on existing individual preventive measures such as LLINs, participants emphasized the complementary nature of larviciding intervention.

“*Larviciding cannot replace other preventive measures such as sleeping under mosquito nets. It’s crucial to use mosquito nets because if you don’t, you’re still at risk of mosquito bites, even if larviciding is being conducted in marshlands, mosquitoes can still breed elsewhere and enter people’s homes. Therefore, larviciding should be seen as complementary to the use of mosquito nets”* (FGD, Female, 39, Bloc 5).

Another FGD participant expressed this view:

*“The use of mosquito nets will remain important because malaria can still return from the few mosquitoes that may stay in our homes. I also request that you provide us with mosquito repellents to hang inside our houses”* (FGD, Male, 51, Bloc 3).

#### Community recommendations for larviciding and malaria control

Community members, drawing from their collective experiences and insights, have articulated a series of comprehensive recommendations aimed at optimizing larviciding and malaria control efforts within their communities. These recommendations encompass various aspects of preventive measures and intervention strategies, reflecting a holistic approach to combatting malaria transmission.

Participants recommended continuing larviciding in marshlands and expanding it to breeding sites near homes:

*“We recommend continuing larviciding in marshlands and expanding it to cover breeding sites around homes”* (FGD, Male, 44, Bloc 4).

They also emphasized the need for regular distribution of long-lasting insecticide-treated nets (LLINs):

*“There should be consistent distribution of long-lasting insecticide-treated nets (LLINs) to ensure households remain protected”* (FGD, Female, 24, Bloc 1).

Some participants called for the return of Indoor Residual Spraying (IRS), noting its past effectiveness:

*“We propose bringing back the practice of Indoor Residual Spraying (IRS) as it was used effectively in the past”* (FGD, Male, 64, Bloc 3).

Participants also stressed the need for better community education on mosquito control:

*“There is a need for more community education and awareness on mosquito control measures to improve participation and understanding”* (FGD, Male, 54, Bloc 4).

FGD participants suggested distributing mosquito repellent lotions through cooperatives to increase protection:

*“We suggest distributing mosquito repellent lotions through our cooperatives to increase personal protection”* (FGD, Female, 36, Bloc 3).

Community members believe that implementing these strategies will significantly aid in eliminating malaria:

*“We firmly believe that by synergizing these initiatives, we can work towards the elimination of malaria”* (FGD, Female, 52, Bloc 4).

## Discussion

Community-based interventions are integral to the success and sustainability of malaria control programmes, particularly in regions where the disease burden remains high [21]. Understanding community perspectives, knowledge, and acceptance of larviciding interventions is crucial for effective implementation and sustained participation [8, 22]. This discussion integrates the findings from the current study conducted in Rwanda with relevant literature to clarify the community factors influencing participation in drone-based larviciding intervention.

The analysis of predictors for willingness to contribute to larviciding efforts revealed important insights into community perceptions and behaviours regarding malaria control. Significant correlations were found between specific occupations, such as rice and vegetable farming and mining, and individuals’ willingness to contribute. Farmers and miners in these sectors likely perceive a direct relationship between their work and increased mosquito exposure due to their frequent proximity to mosquito-prone environments, and many live in the proximity of the marshlands where they work, further heightening their concern about malaria transmission [9].

Frequent exposure to mosquito bites also emerged as a crucial predictor, indicating that personal experiences with the nuisance and health risks posed by mosquitoes’ drive support for larviciding initiatives. This aligns with previous studies from Burkina Faso and Rwanda [8, 23], which emphasize community acceptance and participation in larviciding when individuals recognize the direct impacts of mosquito bites on their health and livelihoods. Moreover, beliefs linking malaria to bites from female Anopheles mosquitoes were strongly correlated with willingness to contribute, reflecting a critical understanding of malaria transmission dynamics. Participants’ recognition of stagnant water as a breeding source for mosquito larvae underscores the necessity of community awareness in promoting effective vector control strategies [24]. The positive association between methods for removing mosquito habitats suggests that community members are inclined to adopt preventive behaviours when they perceive actionable steps to reduce mosquito populations. Additionally, belief in the effectiveness of larviciding, particularly with *Bacillus thuringiensis* var. israelensis (Bti), and confidence in the safety of drone operations further highlights a proactive community approach to health initiatives [22, 25].

The findings of this study underscore a high level of community awareness and acceptance of larviciding intervention. Similar to studies conducted in rural Burkina Faso [8] and Tanzania [24], this research reveals widespread knowledge among participants about malaria transmission and mosquito breeding sites. Participants acknowledged stagnant water bodies as breeding sites for mosquitoes, emphasizing the importance of larval source management in malaria control efforts. Moreover, the community demonstrated a positive attitude towards larviciding, recognizing its effectiveness in reducing mosquito abundance and malaria risk, consistent with findings from previous studies [10, 25, 26].

The acceptance of drone-based larviciding as innovative technology was evident in this study, with participants recognizing the importance of drones in accessing inaccessible areas and facilitating targeted application of larvicides. This aligns with research conducted in Yaoundé, Cameroon [27], which highlighted the efficiency and effectiveness of drones for larval source management. Additionally, the complementary role of hand sprayers in larviciding operations was emphasized by participants, especially large mosquito habitats as well as in areas that are inaccessible to drones. This echoes findings from studies in Kenya and Ethiopia [6], where integrated vector management approaches combining LLINs, larviciding with community mobilization were found to enhance malaria control efforts.

Community engagement emerged as a key determinant of participation in larviciding interventions [28]. This study revealed a strong willingness among community members to contribute financially and physically to larviciding efforts, underscoring their proactive engagement and sense of ownership. Similar findings were reported in studies conducted in Rwanda [2] and Malawi [22], highlighting the importance of community involvement in sustaining malaria control initiatives.

However, the analysis revealed that economic constraints and competing priorities may limit financial contributions, particularly among low-income participants. Addressing these barriers through innovative financing mechanisms, such as subsidies or community cost-sharing models, could enhance participation. The data suggest that leveraging existing community networks and agricultural cooperatives may facilitate broader engagement and scalability. This is consistent with findings regarding the willingness to pay for larviciding interventions, which varied among participants. Factors such as economic constraints significantly influenced contribution levels. This study aligns with prior research in Rwanda [29], where the willingness of rice farmers to engage in larviciding campaigns was assessed. In that study, half of the participants demonstrated a readiness to pay a lump sum of at least 1000 RWF ($1.30) per rice cultivation season. This study found that participants expressed their willingness to contribute approximately 939.52 RWF (equivalent to $0.94) per month.

Participants emphasized the crucial nature of integrating larviciding with established preventive measures such as LLINs, highlighting a holistic approach to malaria prevention and control. This aligns with findings from recent studies [6, 30], which shed light on the potential of larviciding as a complementary strategy in vector control efforts. These studies underscore the importance of integrating larviciding with existing interventions to maximize effectiveness in addressing malaria transmission.

Understanding community preferences and willingness to contribute financially is crucial for designing sustainable and equitable financing mechanisms for larviciding programmes [28]. While this study provides significant insights into community perspectives on drone-based larviciding for malaria control, several limitations should be acknowledged. Firstly, the study was conducted in a specific region of Rwanda, the marshlands of the sub-urban Kigali and focused on agricultural and mining communities potentially limiting the generalizability of findings to other demographic groups with different ecological and socio-economic contexts and may not fully represent the diversity of perspectives across the country. Secondly, the reliance on self-reported data introduces the potential for response bias, as participants may provide socially desirable responses or struggle to accurately recall specific details, particularly regarding their willingness to physically or financially contribute to the intervention. For example, responses to questions about the amount of money or time participants are willing to contribute (monthly financial contributions or labour commitments) may be affected by recall inaccuracies or a tendency to overstate their willingness due to perceived social expectations. Moreover, there is uncertainty about whether individuals’ stated willingness to contribute physically will translate into sustained commitment, given the labour-intensive nature of larviciding, which warrants careful consideration in planning and implementation.

Participants’ willingness to pay may have been influenced by the prevalent notion that public health initiatives are primarily publicly funded, thus not necessitating personal financial contributions. Lastly, the lack of a baseline survey in this research limits the ability to measure changes over time, making it challenging to assess long-term changes in community attitudes and behaviours toward drone-based larviciding interventions. Challenges such as scalability, operational costs, and the need for sustained financing mechanisms require further investigation. Policy frameworks to integrate larviciding into national malaria control strategies should focus on financial sustainability, technical capacity building, and community-driven solutions.

## Conclusion

This study emphasizes the importance of understanding community perspectives and engagement in malaria vector control efforts, specifically focusing on drone-based larviciding intervention. The findings demonstrate a high level of community awareness and acceptance, with participants acknowledging the efficacy of drone-based larviciding in reducing mosquito abundance, densities, and malaria risk. Moreover, the study highlights the crucial role of community engagement in sustaining drone-based larviciding initiatives, evidenced by participants’ willingness to contribute financially and offer unpaid labour.

The research underscores several key advantages of drone-based larviciding, including the ability to complete spraying in a short time with less damage to crops. The targeted application, facilitated by precise mapping of water bodies before spraying Bti, the safety of Bti on crops and humans, particularly on rice and vegetables. Moreover, participants noted a tangible reduction in mosquito nuisance, affording them more time to dedicate to the care of their rice and vegetable crops. Furthermore, participants acknowledged the necessity of complementing drones’ usage with manual sprayers to ensure comprehensive coverage, thereby enhancing the overall effectiveness of the intervention.

Additionally, it is essential to recognize the increased awareness among participants about their role in creating mosquito breeding sites and the need to prevent mosquito bites and malaria transmission. The study also emphasizes the importance of integrated vector management (IVM) for engaging the inters-sectorial collaboration and community participation, and thus, synergistically combining preventive tools to achieve malaria elimination while ensuring sustainability.

Nevertheless, the study acknowledges several limitations and challenges that should be addressed in future drone-based larviciding interventions. The difficulty in assessing long-term changes in community attitudes and behaviours toward drone-based larviciding interventions highlights the need for further research to explore the sustainability of community participation and development of algorithms for targeted larviciding and cost effectiveness. Additionally, the lack of a baseline survey for this research underscores the necessity of comprehensive initial assessments in future studies. Future longitudinal research should include the baseline data collection and development of algorithms that may facilitate the implementation of targeted larviciding, thus explore different options for cost effectiveness and sustainability.

Evaluate the cost-effectiveness of deploying drone-based larviciding interventions by comparing blanket coverage vis- a- vis targeted coverage. This should include a comprehensive analysis of operational costs, scalability, and the intervention’s effectiveness in reducing malaria transmission. Such insights will guide evidence-based decision-making and ensure optimal resource allocation to maximize impact in malaria control strategies.

To ensure long-term sustainability, future drone-based larviciding programmes for malaria vector control should consider developing and implementing innovative financing mechanisms by engaging diverse stakeholders, including local partners such as the Government of Rwanda, international funding agencies, and public–private partnerships (PPPs). These mechanisms should focus on securing sustainable resources to support scalability and programme viability. Additionally, strengthen capacity building through community education initiatives on larviciding to foster greater community ownership and engagement. Prioritize comprehensive training programmes for drone operators and explore local capacity building for maintenance and manufacturing of drones to further ensure the sustainability and cost effectiveness of these interventions.

Overall, the insights gained from this study provide a valuable foundation for improving and scaling drone-based larviciding interventions for malaria control through identification of potential mosquito breeding sites and larviciding. Finally, the results of the study emphasize the importance of community engagement and integrated vector management for sustainable gains in malaria control and its elimination.

## Data Availability

Data are available from the corresponding author upon reasonable request.

## Abbreviations

ANC: Antenatal Care
*Bti*: *Bacillus thuringiensis* Var. israelensis
FGD: Focus Group Discussion
IRS: Indoor residual spraying
ITN: Insecticide-Treated Net
IVM: Integrated Vector Management
LLINs: Long-lasting insecticidal net
LSM: Larval source management
MOH: Ministry of Health
RBC: Rwanda Biomedical Centre
WHO: World Health Organization
